# Stability in the leaf functional traits of understory herbaceous species after 12-yr of nitrogen addition in temperate larch plantations

**DOI:** 10.3389/fpls.2023.1282884

**Published:** 2023-12-05

**Authors:** Tao Yan, Liying Wang, Peilin Wang, Tianyu Zhong

**Affiliations:** ^1^ State Key Laboratory of Herbage Improvement and Grassland Agro-ecosystems, College of Pastoral Agriculture Science and Technology, Lanzhou University, Lanzhou, China; ^2^ Qingyuan Forest CERN, National Observation and Research Station, Shenyang, China

**Keywords:** nitrogen deposition, temperate forest, herbaceous layer, economic and hydraulic traits, stand age

## Abstract

Leaf functional traits play critical roles in plant functioning. Although the functional traits of overstory trees have been extensively studied, minimal research has been conducted regarding understory species, despite the understory layer is an important component of temperate forests. Such insufficiency limit the broader understanding of processes and functions in forest ecosystems, particularly when under the increasing atmospheric nitrogen (N) deposition. Here, we investigated the responses of 18 leaf functional traits in six understory herbaceous species within young and mature stands (three species per stand) in larch (*Larix principis-rupprechtii*) plantations that subjected to 12 years of anthropogenic N addition. We found that N addition did not significantly impact the photosynthetic traits of understory herbaceous species in either stand; it only led to increased chlorophyll content in *Geum aleppicum* Jacq. Similarly, with the exception of decreases in the predawn leaf water potential of *Sanguisorba officinalis* L., N addition did not significantly affect leaf hydraulic traits. With the exception of changes to adaxial epidermis thickness in *Potentilla chinensis* Ser. (decreased) and *G. aleppicum* (increased), N addition had negligible effects on leaf anatomical traits and specific leaf area, however, interspecific variations in the plasticity of leaf anatomical traits were observed. Stable responses to N addition were also observed for nonstructural carbohydrates (NSC) and their components (soluble sugars and starch), with the exception of *Polygonum divaricatum* L., which exhibited increases in NSC. Overall, our results suggest that the functional traits of understory herbaceous species exhibit stability under conditions of long-term N enrichment in temperate plantations.

## 1 Introduction

Atmospheric nitrogen (N) deposition has substantially increased in the past century because of fossil fuel combustion and the use of fertilizers in agriculture (Galloway et al., 2004). Globally, the influence of N deposition on ecosystem structure and function has been extensively studied (Bobbink et al., 2010; Lu et al., 2010a; Marklein and Houlton, 2012; Midolo et al., 2019; Liang et al., 2020). Indeed, N deposition could significantly affect many ecological properties and processes. For example, atmospheric N deposition can lead to soil acidification, imbalances between N and phosphorus (P), the loss of exchangeable base cations, and declines in biodiversity (Bobbink et al., 2010; Lu et al., 2010b; Peñuelas et al., 2013; Lu et al., 2018; Midolo et al., 2019). However, N deposition can also improve plant biomass accumulation and soil carbon sequestration in N-limited ecosystems (Thomas et al., 2010; Maaroufi et al., 2015; Zak et al., 2017; Schulte-Uebbing & de Vries, 2018).

Leaf functional traits, including photosynthetic, hydraulic, and anatomical traits, play critical roles in leaf function, which influence the regulation of plant growth and survival (Wright et al., 2010; Liu et al., 2016; Rowland et al., 2021; Sonawane et al., 2021; Xiong and Flexas, 2022). Increased N availability may affect gas exchange, as well as hydraulic and anatomical structures, with consequences for plant performance (Cai et al., 2017; Báez and Homeier, 2018; Zhang et al., 2021a). Most studies on the responses of functional traits to N addition were focused on grassland species or woody tree species (Liang et al., 2020; Zhang et al., 2021b). For example, Pivovaroff et al. (2016) observed increased predawn water potential (*Ψ*_pd_), but no changes in photosynthesis, leaf hydraulic conductivity (*K*_leaf_), or midday water potential (*Ψ*_md_), in response to long-term N addition in a Mediterranean-type ecosystem. In contrast, Zhang et al. (2021b) reported that short-term N addition increased net photosynthetic rate (*A*_n_) and chlorophyll concentration (Chl), and reduced specific leaf area (SLA) and stomatal conductance (*g*_s_). Additionally, leaf anatomical traits (e.g., palisade mesophyll thickness [PMT], the diameter of the midrib vascular bundle [VBD], and leaf thickness [LT]) can influence leaf photosynthetic and hydraulic capacities; these traits are responsive to environmental changes such as N enrichment (Niinemets et al., 2007; Kröber et al., 2015; Cai et al., 2017; He et al., 2018; Sonawane et al., 2021). The stratum of understory vegetation preserves about 80% of plant biodiversity in temperate forests (Gilliam, 2007). Considering that understory vegetation is an important component of forest ecosystems, functional changes in understory vegetation can substantially affect forest structure and function, such as tree regeneration, carbon-nutrient-water cycling, and stability (Gilliam, 2007; Gilliam et al., 2016; Giuggiola et al., 2018; Landuyt et al., 2019; Blondeel et al., 2020; Xing et al., 2022). For example, in a recent review, Balandier et al. (2022) reported that understory vegetation contributed one-third of ecosystem evapotranspiration in boreal and temperate forests, and the removal of understory vegetation could reduce water competition, with subsequent consequence of increasing soil water content and stimulating sap flow and the growth of overstory tree species (e.g., mean annual radial growth increased by 4.6-fold, Giuggiola et al., 2018). Deng et al. (2016) demonstrated that, when subjected to exogenous N inputs, understory vegetation have competitive advantages in terms of resource acquisition (e.g., P) compared with the corresponding young overstory trees. However, relative to overstory tree species, understory vegetation were largely ignored during the functioning assessments of forest ecosystems, particularly under climate change (Landuyt et al., 2019).

Although suitable levels of N addition can benefit plant productivity, excessive N may weaken or constrain growth (Högberg et al., 2006; Karst et al., 2021), presumably because of nutrient imbalances and changes in functional traits induced by N addition (Högberg et al., 2006; Báez and Homeier, 2018; Li et al., 2022). A recent meta-analysis by Liang et al. (2020) revealed that N addition significantly increases photosynthetic parameters such as photosynthetic rate, *g*_s_, and transpiration rate (E). Moreover, Du et al. (2020) reported that N addition substantially reduces soluble sugars (SS) in woody plant leaves without influencing starch (ST), whereas the opposite effects were observed in herbaceous plant leaves. Notably, the responses of functional traits to climate change have been extensively concentrated on overstory tree species; in contrast, minimal research has been conducted regarding to understory vegetation, particularly with respect to anatomical and hydraulic traits, and moreover, the responses of understory and overstory species may significantly differ (Mo et al., 2020; Jacob et al., 2022). The few studies on understory vegetation showed that N enrichment had limited effects on functional traits such as SLA of forest understory community (Blondeel et al., 2020); and in most cases, long-term (~10-year) N addition exerted little impacts on leaf functional traits of understory vegetation in tropical forests (Mao et al., 2017; Mo et al., 2020). In addition, experimental duration may affect the responses of functional traits to N addition, but most studies have been conducted over relatively short temporal scales (Mao et al., 2018; Yan et al., 2018a; Tang et al., 2021; Zhang et al., 2021b); while studies of the effects of long-term N addition are rare (Blondeel et al., 2020; Liang et al., 2020). Furthermore, stand age may modulate the responses of plants to N addition (Schulte-Uebbing & de Vries, 2018; Yan et al., 2018a), presumably because of differences in environmental conditions. Therefore, analyses of changes in leaf functional traits in response to long-term N addition of understory vegetation under different aged forests would facilitate the comprehensive and predictive changes in forest ecological processes and functions under increasing atmospheric N deposition (Landuyt et al., 2019).

The total area of plantations in the temperate zone has considerably increased in recent decades, particularly in China, which contains approximately one-third of the global plantation area (Payn et al., 2015). Larch (*Larix* spp.) is the main timber tree species in northern China and the Northern Hemisphere (Mason and Zhu, 2014; Yan et al., 2017; Yan et al., 2018b). Here, we selected six understory herbaceous species that occurs in young and mature larch (*L. principis-rupprechtii*) plantations which were subjected to anthropogenic N addition for 12 consecutive years, with the aim to explore how long-term N addition affects the functional traits of understory species. We measured 18 leaf functional traits, including *A*_n_, *g*_s_, E, Chl, *Ψ*_pd_, *Ψ*_md_, *K*_leaf_, adaxial and abaxial epidermis thickness (ADET and ABET), PMT, spongy mesophyll thickness (SMT), VBD, diameter of midrib (MD), LT, SLA, and nonstructural carbohydrates (NSCs) and their components (SS and ST). We hypothesized that long-term N addition would affect leaf photosynthetic, hydraulic, and anatomical traits of understory species because of the typical N-limitation of the study region (Sun et al., 2016). Moreover, we presumed that the magnitude of N-addition-induced changes in functional traits would vary according to species and stand age because of differences in environmental conditions.

## 2 Materials and methods

### 2.1 Study sites

The study was conducted at the Saihanba Ecological Station (42° 25’ N, 117° 15’ E, 1505 m elevation) of Peking University, located in the Saihanba National Forest Park in Hebei Province, northern China. The climate is semi-humid, with long, cold winters (November–March), and short springs and summers. The annual mean temperature is –1.0°C, and the monthly mean ranges from –21.8°C in January to 16.2°C in June. The annual mean precipitation is 460 mm. Snowfall begins in mid-October, and snow melt occurs in early April. Typically, < 30 cm of snow accumulates in winter. Ambient N deposition is 13 kg ha^−1^ year^−1^ (Deng et al., 2016).

### 2.2 Experimental design and sample collection

In 2009, an 11-year-old larch (*Larix principis-rupprechtii*) plantation and a 45-year-old larch plantation were selected to represent young and mature stands, respectively. Both of the stands were fenced by 100 m × 100 m, and each was divided into nine 20 m × 20 m plots, with a buffer zone of approximately 10 m between adjacent plots. Nitrogen addition began in May 2010 and included three treatments: control (no N addition), low N addition (20 kg N ha^−1^ year^−1^; N20), and high N addition (50 kg N ha^−1^ year^−1^; N50). Each treatment was replicated three times. Between May 2010 and the present, liquid urea was applied each month to the N addition treatments during the growing season (May–October) using backpack sprayers to ensure homogeneity within each plot. An equal volume of water was added to the control plots. Details of the experimental design can be seen in Yan et al. (2018c).

In mid-August 2021, we sampled leaves from three understory species in each stand. Specifically, *Vicia sepium* L., *Potentilla chinensis* Ser., and *Polygonum divaricatum* L. were sampled from the young stand; *Agrimonia pilosa* Ldb., *Geum aleppicum* Jacq., and *Sanguisorba officinalis* L. were sampled from the mature stand. For trait measurements, we sampled only fully expanded, mature leaves from healthy individuals with no visible signs of pests or diseases.

### 2.3 Photosynthetic measurements

We selected three individuals of each species with similar growth status in each plot, then sampled fully mature leaves from each individual. Photosynthetic parameters, including *A*_n_, *g*_s_, and E, were measured *in situ* between 09:00 and 11:30 on sunny days with a LI-6800 portable photosynthesis system (Li-Cor Inc., Lincoln, NE, USA). The CO_2_ concentration in the chamber was maintained at 400 μmol mol^−1^, and the temperature of the leaf cuvette was maintained at 25°C. The photosynthetic photon flux density was set at 1500 μmol m^−2^ s^−1^. The relative humidity was maintained at 50–60%. After photosynthesis measurements, Chl was measured on six replicates of each species in each plot using a chlorophyll content meter (CCM-300, Opti-Sciences Inc., Hudson, NH, USA).

We also randomly selected ≥ 5 individuals of each species in each plot for leaf sampling. The leaves were cut with scissors, placed in envelopes inside a cooler, and immediately transported to the laboratory. Leaves were scanned using a digital scanner and leaf area was calculated using ImageJ software. Leaves were then oven-dried at 65°C for ≥ 48 h to obtain leaf dry mass. Specific leaf area (cm^2^ g^−1^) was obtained through the division of leaf area by leaf dry mass.

### 2.4 Leaf hydraulic and anatomical measurements

We collected healthy, intact leaves from five individuals of each species in each plot. The leaf water potential metrics *Ψ*_pd_ and *Ψ*_md_ were measured before sunrise (06:00) and at midday (12:00–14:00), respectively, using a pressure chamber (Model 1505D, PMS Instrument Company, Albany, OR, USA). Immediately after E measurement, the leaves of three individuals were used to measure water potential. Neighboring leaves were covered with aluminum foil for 30 min prior to water potential measurements.

*K*_leaf_ (mmol m^−2^ s^−1^ MPa^−1^) was calculated as:


$$K_{\text{leaf}}=\text{E}/\text{Δ}\Psi$$

where E (mol m^−2^ s^−1^) represents the transpiration rate per unit leaf area as measured with a LI-6800 portable photosynthesis system (Li-Cor Inc.), and Δ*Ψ* (MPa) is the difference in water potential between covered and uncovered leaves.

We assessed leaf anatomy by observing paraffin sections of fresh leaf samples under a light microscope. We sampled five healthy, fully mature leaves from five individuals of each species in each plot. We cut each leaf to extract the leaf blade tissue within 5 mm of either side of the midrib, then sliced this sample into 5 mm segments. Segments were fixed in FAA solution for ≥ 24 h; dehydrated using varying concentrations of alcohol; cleared with xylene; and embedded in paraffin blocks. The paraffin blocks were cut into 5 µm sections using a sliding microtome, stained with safranin-fast green, and sealed with gum. The prepared cross-sections were observed under a light microscope. ImageJ software was used to measure anatomical traits including adaxial epidermis thickness, abaxial epidermis thickness, palisade mesophyll thickness, spongy mesophyll thickness, diameter of midrib, diameter of the midrib vascular bundle, and leaf thickness.

### 2.5 NSC measurements

NSCs were determined using the modified anthrone method. Fresh leaf samples were microwaved at 700 W for 90 s to stop enzymatic activity, then oven-dried for ≥ 48 h at 65°C to achieve a constant weight and ground with a ball mill (MM400, RETSCH GmbH, Haan, Germany). Powdered leaf samples (0.1 g) were placed into 10 ml centrifuge tubes with 2 ml of 80% ethanol. The mixture was incubated for 30 min in a water bath at 80°C, then centrifuged at 3500 rpm for 10 min. The supernatant was retained, and the residue was extracted two additional times for SS measurement. After SS extraction, the residue was subjected to ST extraction by adding 2 ml of distilled water and boiling the solution in a ~100°C water bath for 15 min, then adding 2 ml of 9.2 mol L^−1^ HClO_4_ and shaking the mixture for 15 min. Then, 4 ml of distilled water was added and the solution was centrifuged at 3500 rpm for 10 min. Subsequently, 2 ml of 4.6 mol L^−1^ HClO_4_ and 5 ml of distilled water were added to the precipitate, the solution was centrifuged at 3500 rpm for 5 min, and the supernatant was retained. Twice, we added an 5 ml of distilled water to wash the residue, then combined it into the supernatant. Concentrations of SS and ST were measured using a UV-Vis spectrophotometer with an absorbance of 620 nm. Total NSC concentrations were calculated by summing SS and ST.

### 2.6 Statistical analyses

Two-way analysis of variance was used to determine how functional traits were affected by N addition, species, and the interaction between those two factors. Least significant difference tests were used to assess differences among treatments. When necessary, the data were transformed to improve homogeneity of variance and/or normality. When the transformed data did not satisfy these assumptions, non-parametric Kruskal–Wallis tests were used. All analyses were conducted using SPSS 16.0 for Windows (SPSS Inc., Chicago, IL, USA), and differences were considered statistically significant at *P*-values< 0.05.

## 3 Results

### 3.1 Photosynthetic traits

**Figure 1:**
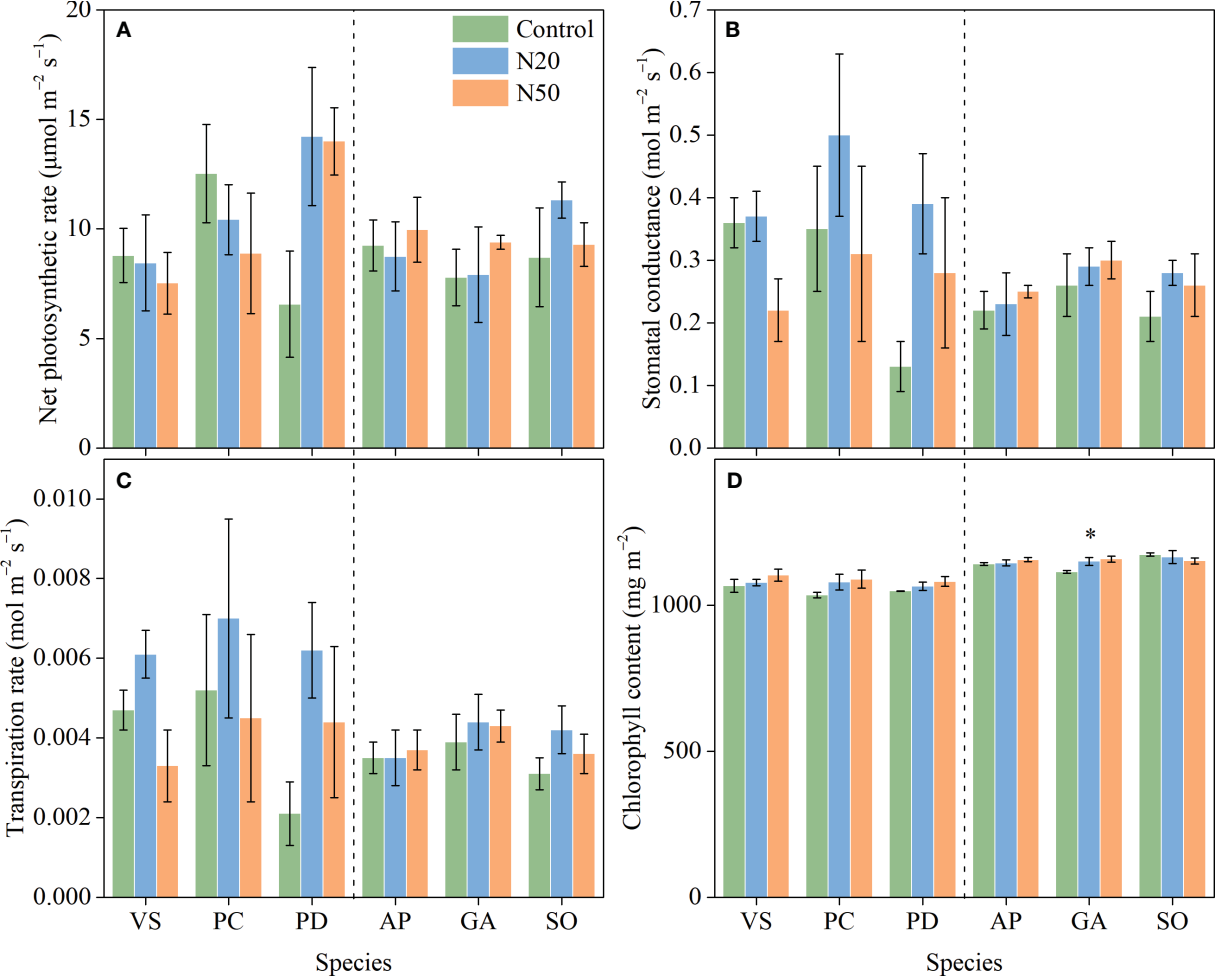
Effects of N addition on *A*_n_ **(A)**, *g*_s_ **(B)**, E **(C)**, and Chl **(D)** of *Vicia sepium* (VS), *Potentilla chinensis* (PC), *Polygonum divaricatum* (PD) in the young stand, and *Agrimonia pilosa* (AP), *Geum aleppicum* (GA), and *Sanguisorba officinalis* (SO) in the mature stand. Asterisk represents significant differences among N addition treatments (*P* < 0.05). Values are means ± standard errors (n = 3).

Long-term N addition did not significantly impact *A*_n_, *g*_s_, or E in any of the six understory species in either stand (**Figure 1**); however, Chl was increased in *G. aleppicum*. We observed minimal variation in photosynthetic traits among species (**Table 1**).

**Table 1:** Results (*P-*values) of two-way analyses of variance.

Traits	Young			Mature		
	N	Species	N × Species	N	Species	N × Species
*A*_n_	0.619	0.176	0.108	0.700	0.498	0.713
*g*_s_	0.113	0.317	0.562	0.388	0.262	0.918
E	0.123	0.582	0.789	0.446	0.318	0.913
Chl	**0.050**	0.485	0.921	0.365	0.062	0.080
*Ψ*_pd_	0.114	0.509	0.199	0.528	**0.010**	0.338
*Ψ*_md_	0.064	**0.010**	0.977	0.565	0.916	0.430
*K*_leaf_	0.233	0.457	0.887	0.324	0.911	0.948
ADET	0.194	**<0.001**	0.817	0.462	**<0.001**	0.396
ABET	0.552	**<0.001**	0.897	0.730	**<0.001**	0.333
PMT	0.481	**<0.001**	0.774	**0.024**	**<0.001**	0.403
SMT	0.928	**<0.001**	0.951	0.706	0.072	0.458
MD	0.097	**0.032**	0.688	0.654	**<0.001**	0.953
VBD	0.208	**<0.001**	0.294	0.745	**0.009**	0.932
LT	0.946	**<0.001**	0.875	0.464	**<0.001**	0.215
SLA	0.679	0.340	0.428	**0.045**	0.210	0.286
NSC	**0.045**	**<0.001**	0.138	0.097	**<0.001**	0.704
SS	0.434	**<0.001**	0.190	0.814	**<0.001**	0.102
ST	0.051	**<0.001**	**0.020**	0.081	**<0.001**	0.466

*A*_n_, net photosynthetic rates; *g*_s_, stomatal conductance; E, transpiration rate; Chl, chlorophyll concentration; *Ψ*_pd_, predawn water potential; *Ψ*_md_, midday water potential; *K*_leaf_, leaf hydraulic conductivity; ADET, adaxial epidermis thickness; ABET, abaxial epidermis thickness; PMT, palisade mesophyll thickness; SMT, spongy mesophyll thickness; MD, diameter of midrib; VBD, diameter of the midrib vascular bundle; LT, leaf thickness; SLA, specific leaf area; NSC, nonstructural carbohydrate; SS, soluble sugars; and ST, starch. Bold text indicates significant differences (*P* < 0.05).

### 3.2 Leaf hydraulic traits

**Figure 2:**
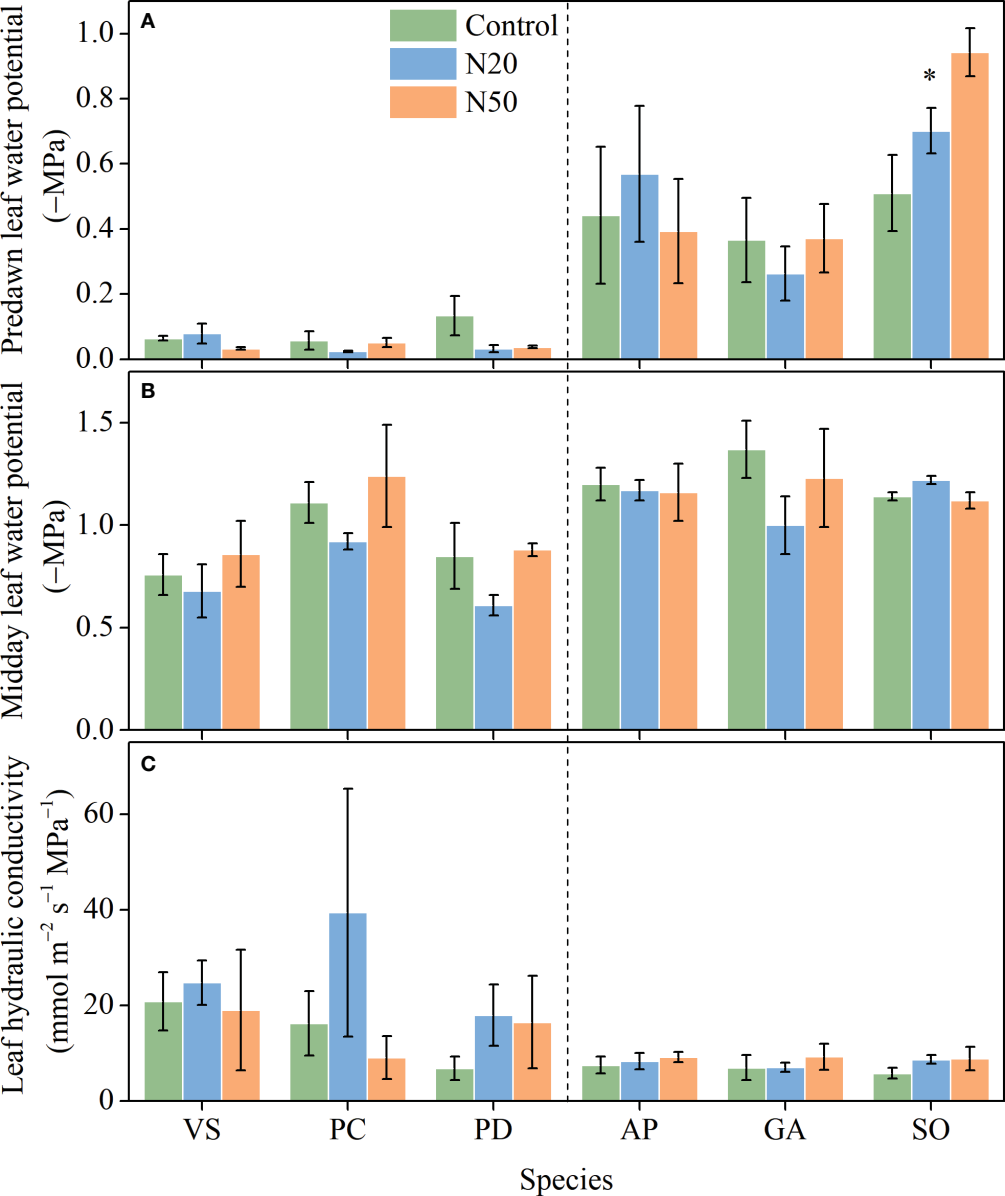
Effects of N addition on *Ψ*_pd_ **(A)**, *Ψ*_md_ **(B)**, and *K*_leaf_ **(C)** of *Vicia sepium* (VS), *Potentilla chinensis* (PC), *Polygonum divaricatum* (PD) in the young stand, and *Agrimonia pilosa* (AP), *Geum aleppicum* (GA), and *Sanguisorba officinalis* (SO) in the mature stand. Asterisk represents significant differences among N addition treatments (*P* < 0.05). Values are means ± standard errors (n = 3).

With the exception of a reduction in *Ψ*_pd_ in *S. officinalis*, N addition did not significantly affect *Ψ*_pd_, *Ψ*_md_, or *K*_leaf_ in any of the six species (**Figure 2**). *Ψ*_pd_ and *Ψ*_md_ significantly varied among species in the mature and young stands, respectively, but *K*_leaf_ had no variation among species in either stand (**Table 1**).

### 3.3 Leaf anatomical traits

**Figure 3:**
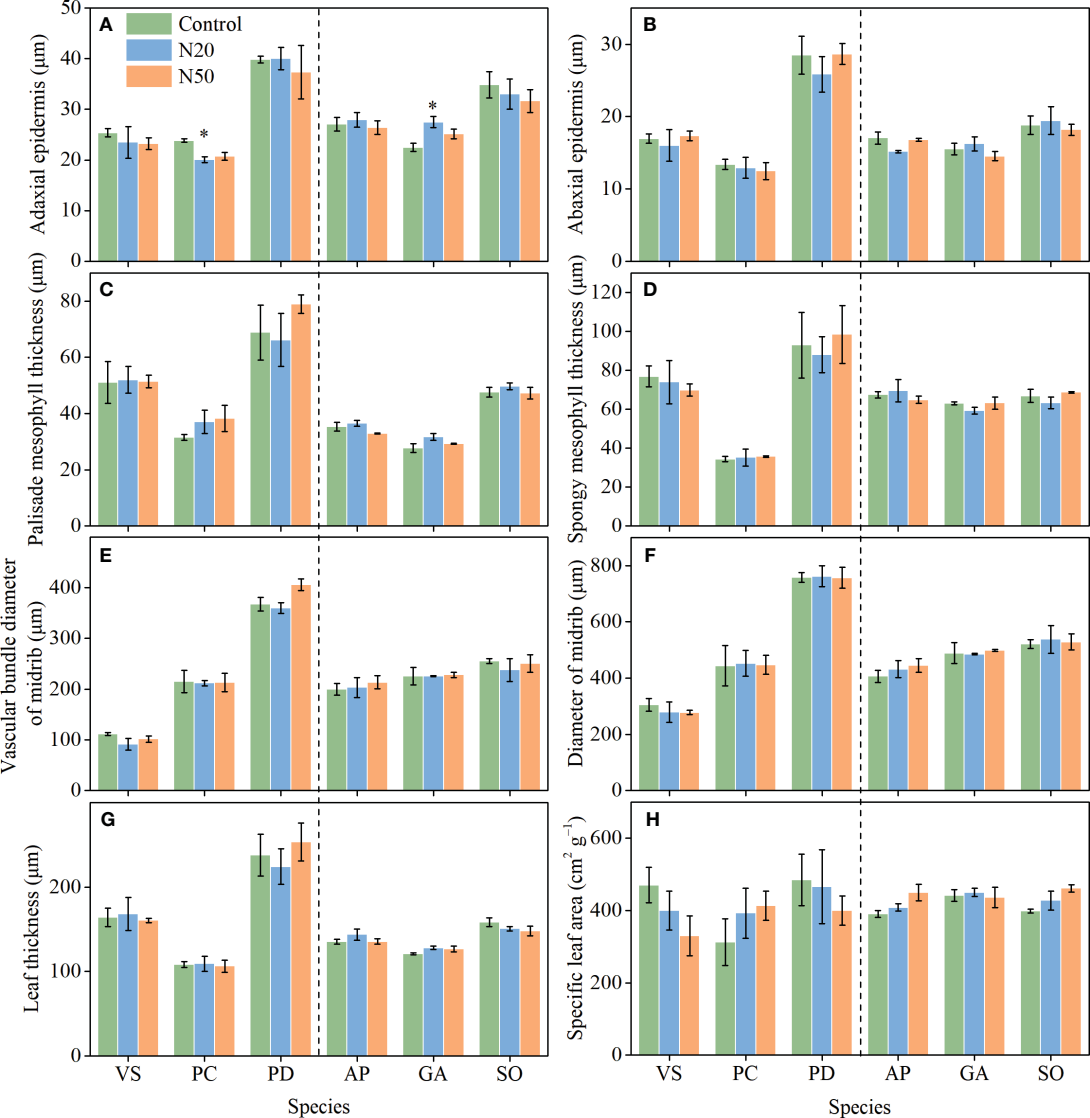
Effects of N addition on ADET **(A)**, ABET **(B)**, PMT **(C)**, SMT **(D)**, VBD **(E)**, MD **(F)**, LT **(G)**, and SLA **(H)** of *Vicia sepium* (VS), *Potentilla chinensis* (PC), *Polygonum divaricatum* (PD) in the young stand, and *Agrimonia pilosa* (AP), *Geum aleppicum* (GA), and *Sanguisorba officinalis* (SO) in the mature stand. Asterisk represents significant differences among N addition treatments (*P* < 0.05). Values are means ± standard errors (n = 3).

After N addition, adaxial epidermis thickness decreased in *P. chinensis* in the young stand, whereas it increased in *G. aleppicum* in the mature stand (**Figure 3**). Nitrogen addition had no other significant impacts on leaf anatomical traits. However, with the exception of SLA, leaf anatomical traits significantly differed among species; spongy mesophyll thickness slightly differed among species in the mature stand (*P* = 0.072).

### 3.4 NSC concentrations

**Figure 4:**
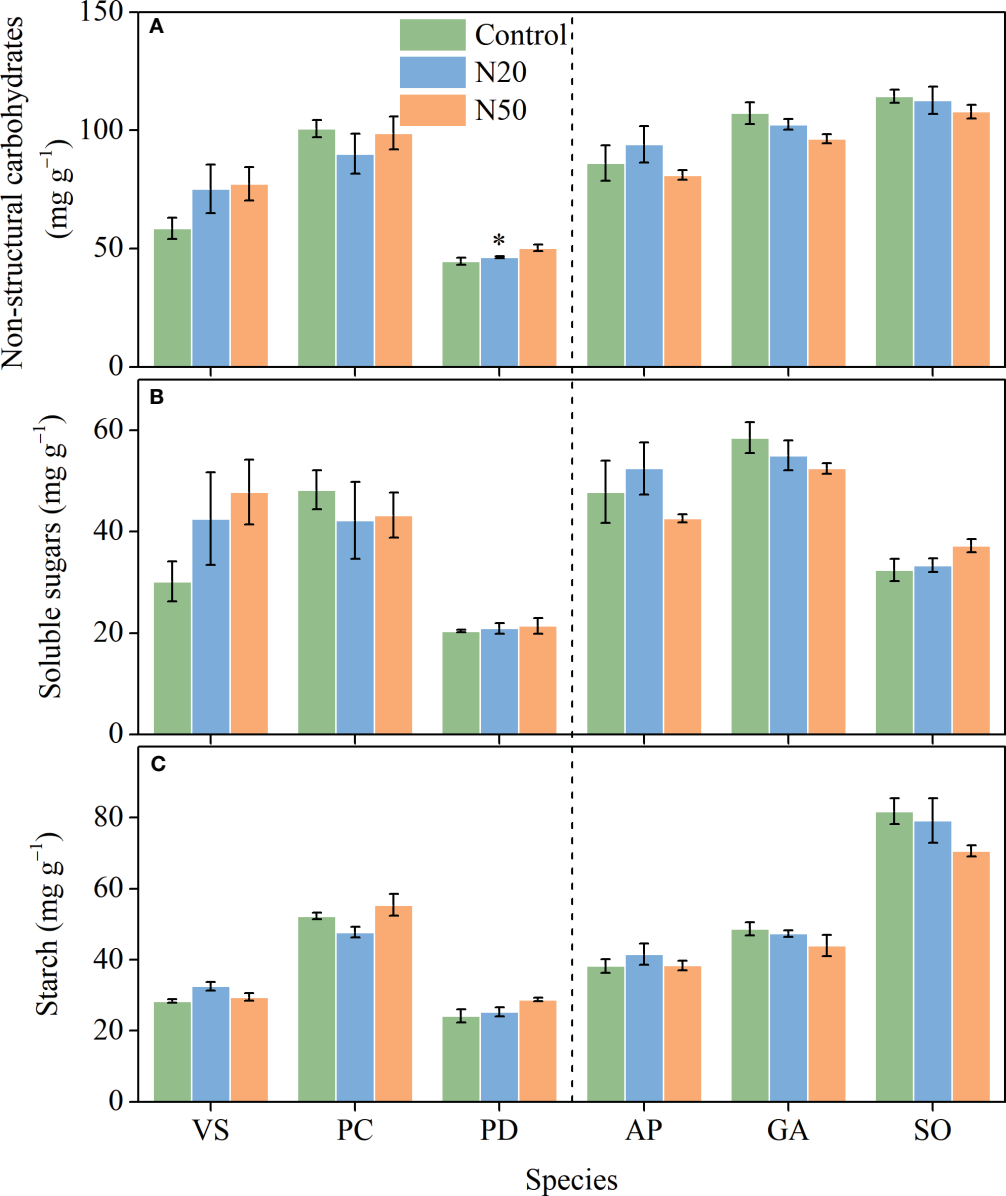
Effects of N addition on NSC **(A)**, SS **(B)**, and ST **(C)** of *Vicia sepium* (VS), *Potentilla chinensis* (PC), *Polygonum divaricatum* (PD) in the young stand, and *Agrimonia pilosa* (AP), *Geum aleppicum* (GA), and *Sanguisorba officinalis* (SO) in the mature stand. Asterisk represents significant differences among N addition treatments (*P* < 0.05). Values are means ± standard errors (n = 3).

With the exception of significant increases in total NSC concentrations in *P. divaricatum* in the young stand (**Figure 4**), neither NSC nor its components (SS and ST) exhibited significant responses to N addition in any understory species. However, we observed significant interspecific variation in all three variables.

## 4 Discussion

### 4.1 Effects of long-term N addition on photosynthetic parameters

Numerous studies have revealed positive correlations between leaf N concentrations and photosynthetic capacity (Evans, 1989; Reich et al., 1997; Wright et al., 2004), largely because N is an important component of photosynthetic proteins (Evans, 1989; Güsewell, 2004; Evans and Clarke, 2019). Contrary to our hypothesis, we did not observe improvements in the *A*_n_ of understory species after long-term N addition (**Figure 1**). However, N addition had significant positive effects on leaf N concentrations of understory species in the mature stand (Yan et al., 2018a). These findings indicate that N addition-induced increases in leaf N concentrations do not necessarily lead to increases in photosynthetic carbon assimilation capacity, probably due to that larger fraction of leaf N was allocated to cell walls but not photosynthetic proteins (Bauer et al., 2004; Talhelm et al., 2011; Pivovaroff et al., 2016; Onoda et al., 2017). Our results differ from the findings in other N-limited ecosystems, where higher N availability typically improves leaf physiological traits (Cooke et al., 2005; Zhang et al., 2021a,b).

The lack of changes in *A*_n_ in response to long-term N enrichment can be attributed to various mechanisms. Nitrogen is unlikely to be a key limiting factor for understory species in the young stand; thus, N addition may have induced few changes in leaf N and had minimal effects on the allocation of N to photosynthetic processes (Yan et al., 2018a). However, the availability of other resources, such as light, may constrain the growth of understory species (Yan et al., 2018a; De Pauw et al., 2022); in the mature stand, increased leaf N concentrations were presumably insufficient to compensate for the negative effects of increased overstory shading on photosynthesis (Palmroth et al., 2014; Yan et al., 2018a). Additionally, the excess N in leaves may have been allocated to soluble proteins and/or free amino acids, rather than chlorophyll (Mao et al., 2018). Since NSCs are the primary products of photosynthesis (Hartmann and Trumbore, 2016), and thus, NSCs did not vary (**Figure 4**) due to the relatively stable photosynthetic rates. The exception of the statistically significant increase in NSCs observed in *P. divaricatum* can presumably be attributed to corresponding improvements in leaf photosynthesis albeit not significant (**Figures 1**, **4**).

With the exception of *G. aleppicum*, N addition had no impact on Chl among species in the mature stand (**Figure 1**) despite increased leaf N concentrations (Yan et al., 2018a). This result implies that the additional leaf N was probably incorporated into soluble proteins and/or free amino acids, rather than chlorophyll; however, further investigations into leaf N metabolism are needed to confirm this hypothesis.

### 4.2 Effects of long-term N addition on leaf hydraulic traits

Leaf water transport plays a critical role in regulating the growth and survival of plants, particularly in the context of increasing atmospheric N deposition (Sack and Holbrook, 2006; Wang et al., 2016; Jin et al., 2020). The efficiency of water transport through the leaf can be quantified using *K*_leaf_ (Sack and Holbrook, 2006). Given that water transport resistance is generally higher in leaves than in stems and shoots, *K*_leaf_ has a substantial impact on water transport at the whole-plant scale (Sack et al., 2003; Sack et al., 2005; Wang et al., 2016), particularly in understory herbaceous species. Analyses of trees have shown that N addition significantly affects *K*_leaf_, thereby influencing whole-plant water use (Wang et al., 2016; Jin et al., 2020; Zhang et al., 2021c). However, in contrast to our hypotheses, N addition did not alter *K*_leaf_ values in understory species in either stand (**Figure 2**). The most probable explanation for this result arises from the lack of changes in leaf anatomical structure, such as VBD (**Figure 3**). Leaf anatomical traits (e.g., VBD) determine *K*_leaf_, and plants with larger VBDs are generally characterized by higher *K*_leaf_ values (Sack and Holbrook, 2006; Sonawane et al., 2021; Xiong and Flexas, 2022). Accordingly, the lack of changes in *g*_s_ and E (**Figure 1**) is understandable.

Leaf water potential may reflect the functional capacity of plants to respond to water availability. Among leaf water metrics, *Ψ*_pd_ is considered the most useful and representative one for determining whether plants suffer from drought stress (Trugman et al., 2021). With the exception of a reduction in *Ψ*_pd_ in *S. officinalis* (**Figure 2**), *Ψ*_pd_ and *Ψ*_md_ remained relatively constant in both stands despite long-term N inputs. These results indicate that although *S. officinalis* may experience drought stress, long-term N addition had minor impacts on leaf water relations in the other understory species.

### 4.3 Effects of long-term N addition on leaf anatomical traits

Leaf anatomical traits are associated with leaf economics (e.g., photosynthetic rate) and leaf hydraulics (e.g., hydraulic conductivity) (Niinemets et al., 2007; Liang et al., 2019; Sonawane et al., 2021; Xiong and Flexas, 2022). For example, a thicker palisade mesophyll layer may contain more chloroplasts, thereby optimizing photosynthesis; in contrast, a thinner epidermis may promote gas exchange and increase transpiration (Kröber et al., 2015; He et al., 2018). Thus, in the context of environmental change (e.g., increased N availability), we expect plants to exhibit adaptations in leaf anatomy that would maximize overall plant function (Chen et al., 2010; Cai et al., 2017; He et al., 2018). Previous studies have demonstrated that even short-term N addition affects some leaf anatomical traits, such as the diameters of the midrib and midrib vascular bundle in *Arabidopsis thaliana*, without affecting other traits (e.g., thicknesses of leaves, palisade and spongy mesophyll layers, and adaxial and abaxial epidermis) (Cai et al., 2017). Contrary to our hypotheses, however, few of the measured leaf anatomical traits exhibited changes despite 12 years of N addition (**Figure 3**). The apparent stability of leaf anatomical traits reflects the acclimation of understory species to long-term N inputs in temperate larch plantations. Our findings differ from the results of Zhang et al. (2021c), which showed that two tree species (*Castanopsis chinensis* and *Schima superba*) in a subtropical forest exhibited decreased leaf thickness after 6-year canopy N addition, and Guo et al. (2022), which reported increases in palisade mesophyll thickness, leaf thickness, and palisade-spongy mesophyll ratio for the herbaceous species of *Stipa glareosa* in a desert steppe after 3-year N addition. Furthermore, we observed substantial interspecific variations in leaf anatomical traits (**Figure 3**; **Table 1**), indicating that these understory species exhibited distinct adaptive strategies.

Nevertheless, we selected only 3–5 individuals per species within each plot, which may, to some extent, limit the statistical power, thereby, more replicates might be better to detect the differences. Furthermore, trait correlations and other environmental factors such as temperature, drought, and herbivore outbreaks, may have constrained the responses of leaf functional traits to N addition, which deserve further investigations.

## 5 Conclusion

Using a 12-year anthropogenic N addition experiment, we investigated the responses of 18 leaf functional traits in six understory herbaceous species to increased N availability in young and mature larch plantations in northern China. Contrary to our hypotheses, understory species exhibited few eco-physiological changes in response to long-term N addition. This lack of response highlights the capacity of understory species to maintain the stability of functional traits. Confirmation of the representativeness of our results requires further investigations into the plasticity of other traits and species in these larch plantations.

## Data availability statement

The raw data supporting the conclusions of this article will be made available by the authors, without undue reservation.

## Author contributions

TY: Conceptualization, Formal Analysis, Funding acquisition, Investigation, Methodology, Project administration, Supervision, Validation, Visualization, Writing – original draft. LW: Data curation, Formal Analysis, Investigation, Methodology, Visualization. PW: Investigation, Methodology. TZ: Investigation, Methodology.
